# Correction: Use of sedative-hypnotics and the risk of Alzheimer’s dementia: A retrospective cohort study

**DOI:** 10.1371/journal.pone.0206094

**Published:** 2018-10-16

**Authors:** The PLOS ONE Staff

## Notice of Republication

This article was republished on October 5, 2018 to correct for an error to the author byline introduced during the typesetting process. The publisher apologizes for the error. Please download this article again to view the correct version. The originally published, uncorrected article and the republished, corrected article are provided here for reference.

## Supporting information

S1 File Originally published, uncorrected article. (PDF)

S2 File Republished, corrected article. (PDF)
