# Supplementary material for: Use of sedative-hypnotics and the risk of Alzheimer’s dementia: A retrospective cohort study
Source: PLoS One. 2018 Sep 24;13(9):e0204413. doi: 10.1371/journal.pone.0204413 (PMC6152975; doi:10.1371/journal.pone.0204413)
Supplement: S1 Appendix — (DOCX) [file pone.0204413.s001.docx]

**S1 Appendix. The list of study drugs in this study**

| Drug name | Class | ATC code |
| --- | --- | --- |
| Etizolam | Short-acting benzodiazepine | N05BA |
| Mexazolam | Short-acting benzodiazepine | N05BA |
| Triazolam | Short-acting benzodiazepine | N05CD |
| Alprazolam | Intermediate-acting benzodiazepine | N05BA |
| Bromazepam | Intermediate-acting benzodiazepine | N05BA |
| Clonazepam | Intermediate-acting benzodiazepine | N03AE |
| Clotiazepam | Intermediate-acting benzodiazepine | N05BA |
| Lorazepam | Intermediate-acting benzodiazepine | N05BA |
| Clobazam | Long-acting benzodiazepine | N05BA |
| Clorazepate | Long-acting benzodiazepine | N05BA |
| Diazepam | Long-acting benzodiazepine | N05BA |
| Flunitrazepam | Long-acting benzodiazepine | N05CD |
| Flurazepam | Long-acting benzodiazepine | N05CD |
| Zolpidem | Zolpidem | N05CF |
| Amitriptyline | Sedative antidepressants | N06AA |
| Imipramine | Sedative antidepressants | N06AA |
| Mirtazapine | Sedative antidepressants | N06AX |
| Nortryptiline | Sedative antidepressants | N06AA |
| Trazodone | Sedative antidepressants | N06AX |
| Chlorpromazine | Sedative low potent antipsychotics | N05AA |
| Levomepromazine | Sedative low potent antipsychotics | N05AA |
| Quetiapine | Sedative low potent antipsychotics | N05AH |

Anatomical Therapeutic Chemical Classification System; ATC
