# Supplementary material for: Use of sedative-hypnotics and the risk of Alzheimer’s dementia: A retrospective cohort study
Source: PLoS One. 2018 Sep 24;13(9):e0204413. doi: 10.1371/journal.pone.0204413 (PMC6152975; doi:10.1371/journal.pone.0204413)
Supplement: S1 Table — (DOCX) [file pone.0204413.s002.docx]

**S1 Table. The hazard ratios of all covariates on the risk of Alzheimer’s dementia**

| **Covariates** | **Person-years** | **Number of events** | **Crude HR** | **95% CI** | **Adjusted HR*** | **95% CI** | **Adjusted HR†** | **95% CI** |
| --- | --- | --- | --- | --- | --- | --- | --- | --- |
| **Sex** |  |  |  |  |  |  |  |  |
| **Male** | 1,590,492 | 11,579 | 1.00 | Reference | 1.00 | Reference | 1.00 | Reference |
| **Female** | 1,351,596 | 16,346 | 1.15 | (1.12-1.18) | 1.18 | (1.15-1.20) | 1.17 | (1.14-1.19) |
| **Insurance preimim** |  |  |  |  |  |  |  |  |
| **Low** | 1,069,709 | 11,999 | 1.00 | Reference | 1.00 | Reference | 1.00 | Reference |
| **Middle** | 940,439 | 7,436 | 0.85 | (0.82-0.87) | 0.86 | (0.83-0.88) | 0.87 | (0.84-0.89) |
| **High** | 931,939 | 8,490 | 0.79 | (0.77-0.81) | 0.80 | (0.78-0.82) | 0.81 | (0.78-0.83) |
| **Comorbidities‡** |  |  |  |  |  |  |  |  |
| **Diabetes mellitus** | 599,691 | 6,256 | 1.23 | (1.19-1.26) | 1.30 | (1.26-1.34) | 1.29 | (1.25-1.33) |
| **Hypertension** | 1,411,845 | 14,620 | 0.94 | (0.92-0.96) | 0.85 | (0.83-0.87) | 0.84 | (0.82-0.86) |
| **Hyperlipidemia** | 681,673 | 5,130 | 0.90 | (0.87-0.92) | 0.79 | (0.77-0.82) | 0.78 | (0.75-0.80) |
| **Cerebrovascular disease** | 227,946 | 4,695 | 1.77 | (1.71-1.82) | 1.87 | (1.81-1.93) | 1.78 | (1.72-1.84) |
| **Insomnia** | 86,655 | 1,433 | 1.39 | (1.31-1.46) | 0.00 | (0.00-0.00) | 1.08 | (1.02-1.14) |
| **Anxiety disorders** | 193,614 | 2,990 | 1.40 | (1.35-1.45) | 0.00 | (0.00-0.00) | 1.18 | (1.13-1.23) |
| **Depression** | 84,782 | 1,761 | 2.06 | (1.96-2.16) | 0.00 | (0.00-0.00) | 1.68 | (1.59-1.77) |
| **Psychotic disorders** | 15,165 | 362 | 3.94 | (3.55-4.38) | 0.00 | (0.00-0.00) | 3.02 | (2.72-3.36) |

Defined daily dose; DDD

*adjusted for sex, diabetes mellitus, hypertension, hyperlipidaemia, cerebrovascular disease, income level

†adjusted for sex, diabetes mellitus, hypertension, hyperlipidaemia, cerebrovascular disease, income level, anxiety, insomnia, depression, psychotic disorder

‡Hazard ratios of each comorbidities were calculated compared to disease free subject.
