# Supplementary material for: Use of sedative-hypnotics and the risk of Alzheimer’s dementia: A retrospective cohort study
Source: PLoS One. 2018 Sep 24;13(9):e0204413. doi: 10.1371/journal.pone.0204413 (PMC6152975; doi:10.1371/journal.pone.0204413)
Supplement: S2 Table — (DOCX) [file pone.0204413.s003.docx]

S2 Table. Hazard ratios of use of sedative-hypnotics on Alzheimer’s dementia among subjects who had insomnia

|  | **Person-years** | **Number of events** | **Crude HR** | **95% CI** | **Adjusted HR*** | **95% CI** | **Adjusted HR**† | **95% CI** |
| --- | --- | --- | --- | --- | --- | --- | --- | --- |
| **<30 DDD of any sedative-hypnotics** | 74,950 | 885 | 1.00 | Reference | 1.00 | Reference | 1.00 | Reference |
| **≥30 DDD of any sedative-hypnotics** | 11,705 | 548 | 2.28 | (2.03 - 2.55) | 2.30 | (2.06 - 2.58) | 2.31 | (2.06 - 2.59) |
|  |  |  |  |  |  |  |  |  |
| **<30 DDD of any sedative-hypnotics** | 74,950 | 885 | 1.00 | Reference | 1.00 | Reference | 1.00 | Reference |
| **30-179 DDD of any sedative-hypnotics** | 6,848 | 263 | 1.70 | (1.45 - 1.98) | 1.74 | (1.49 - 2.03) | 1.77 | (1.51 - 2.06) |
| **180-359 DDD of any sedative-hypnotics** | 2,203 | 118 | 1.54 | (1.19 - 1.98) | 1.55 | (1.20 - 2.00) | 1.57 | (1.21 - 2.03) |
| **≥360 DDD of any sedative-hypnotics** | 2,654 | 167 | 2.50 | (2.07 - 3.01) | 2.49 | (2.07 - 3.01) | 2.50 | (2.07 - 3.02) |
|  |  |  |  |  |  |  |  |  |
| **<30 DDD of GABAA‡ and other drugs§** | 75,104 | 892 | 1.00 | Reference | 1.00 | Reference | 1.00 | Reference |
| **≥30 DDD of GABAA** | 9,928 | 459 | 2.15 | (1.91 - 2.43) | 2.19 | (1.94 - 2.47) | 2.22 | (1.97 - 2.51) |
| **≥30 DDD of other drugs** | 426 | 5 | 0.71 | (0.29 - 1.71) | 0.70 | (0.29 - 1.68) | 0.68 | (0.28 - 1.65) |
| **≥30 DDD of GABAA and ≥30 DDD of other drugs** | 1,198 | 77 | 2.98 | (2.31 - 3.84) | 2.94 | (2.28 - 3.80) | 2.81 | (2.17 - 3.64) |
|  |  |  |  |  |  |  |  |  |
| **<30 DDD of benzodiazepines** | 75,932 | 926 | 1.00 | Reference | 1.00 | Reference | 1.00 | Reference |
| **≥30 DDD of short-acting benzodiazepines** | 1,243 | 58 | 1.64 | (1.21 - 2.23) | 1.74 | (1.28 - 2.36) | 1.81 | (1.33 - 2.46) |
| **≥30 DDD of intermediate-acting benzodiazepines** | 1,872 | 74 | 1.48 | (1.11 - 1.97) | 1.52 | (1.14 - 2.03) | 1.54 | (1.15 - 2.06) |
| **≥30 DDD of long-acting benzodiazepines** | 2,186 | 115 | 2.20 | (1.78 - 2.73) | 2.21 | (1.78 - 2.73) | 2.26 | (1.82 - 2.81) |

Defined daily dose; DDD

*adjusted for sex, diabetes mellitus, hypertension, hyperlipidaemia, cerebrovascular disease, insurance premium

†adjusted for sex, diabetes mellitus, hypertension, hyperlipidaemia, cerebrovascular disease, insurance premium, anxiety, depression, psychotic disorder ‡benzodiazepines or zolpidem

§antidepressants or low-dose antipsychotic
