# Supplementary material for: Use of sedative-hypnotics and the risk of Alzheimer’s dementia: A retrospective cohort study
Source: PLoS One. 2018 Sep 24;13(9):e0204413. doi: 10.1371/journal.pone.0204413 (PMC6152975; doi:10.1371/journal.pone.0204413)
Supplement: S3 Table — (DOCX) [file pone.0204413.s004.docx]

**S3 Table. The hazard ratios of use of sedative-hypnotics on Alzheimer’s dementia based on dosage**

|  | **Person-years** | **Number of events** | **Crude HR** | **95% CI** | **Adjusted HR*** | **95% CI** | **Adjusted HR†** | **95% CI** |
| --- | --- | --- | --- | --- | --- | --- | --- | --- |
| **none of any sedative-hypnotics** | 2,411,991 | 17,144 | 1.00 | Reference | 1.00 | Reference | 1.00 | Reference |
| **1-29 DDD of any sedative-hypnotics** | 427,770 | 7,446 | 1.49 | (1.45-1.54) | 1.53 | (1.49-1.58) | 1.54 | (1.50-1.58) |
| **30-179 DDD of any sedative-hypnotics** | 80,439 | 2,371 | 1.95 | (1.86-2.04) | 1.96 | (1.87-2.06) | 1.92 | (1.83-2.01) |
| **180-359 DDD of any sedative-hypnotics** | 12,575 | 526 | 2.05 | (1.84-2.27) | 2.00 | (1.80-2.22) | 1.89 | (1.70-2.10) |
| **≥360 DDD of any sedative-hypnotics** | 9,312 | 438 | 2.47 | (2.22-2.75) | 2.36 | (2.12-2.63) | 2.12 | (1.89-2.36) |

Defined daily dose; DDD

*adjusted for sex, diabetes mellitus, hypertension, hyperlipidaemia, cerebrovascular disease, insurance premium

†adjusted for sex, diabetes mellitus, hypertension, hyperlipidaemia, cerebrovascular disease, insurance premium, anxiety, insomnia, depression, psychotic disorder
