# Supplementary material for: Correction: Use of sedative-hypnotics and the risk of Alzheimer’s dementia: A retrospective cohort study
Source: PLoS One. 2018 Oct 16;13(10):e0206094. doi: 10.1371/journal.pone.0206094 (PMC6191145; doi:10.1371/journal.pone.0206094)
Supplement: S1 File — (PDF) [file pone.0206094.s001.pdf]

PLOS ONE

RESEARCH ARTICLE

Use of sedative-hypnotics and the risk of Alzheimer's dementia: A retrospective cohort study

Joonki Lee[1], Sun Jae Jung[2†], Jae-won Choi[3], Aesun Shin[1]*, Yu Jin Lee[4]*

1 Department of Preventive Medicine, Seoul National University College of Medicine, Seoul, Republic of Korea, **2** Department of Epidemiology, Harvard T.H.Chan School of Public Health, Boston, MA, United States of America, **3** Department of Neuropsychiatry, Eulji University School of Medicine, Eulji General Hospital, Seoul, Republic of Korea, **4** Department of Psychiatry and Center for Sleep and Chronobiology, Seoul National University College of Medicine, Seoul, Republic of Korea

† Deceased.
* shinaesun@snu.ac.kr (AS); ewpsyche@snu.ac.kr (YJL)

Check for updates

OPEN ACCESS

Citation: Lee J, Jung SJ, Choi J-w, Shin A, Lee YJ (2018) Use of sedative-hypnotics and the risk of Alzheimer's dementia: A retrospective cohort study. PLoS ONE 13(9): e0204413. https://doi.org/10.1371/journal.pone.0204413

Editor: Andrea Romigi, University of Rome Tor Vergata, ITALY

Received: March 19, 2018

Accepted: September 9, 2018

Published: September 24, 2018

Copyright: © 2018 Lee et al. This is an open access article distributed under the terms of the Creative Commons Attribution License, which permits unrestricted use, distribution, and reproduction in any medium, provided the original author and source are credited.

Data Availability Statement: Data are available from the National Health Insurance Sharing Service of Korea. KNHISS does not allow researchers to provide data personally or share publicly and therefore, the authors cannot provide the data. However, all researchers can access the data in the same manner as the authors upon completing the online data request form. Access to NHIS-NHC data can be achieved from the website of NHISS (https://nhiss.nhis.or.kr) after promising to follow the research ethics through the website and completing the application process (http://nhiss.nhis.or.kr/bd/ab/bdaba021eng.do). After receiving

Abstract

There has been a growing interest in the relationship between sedative-hypnotics use and the risk of Alzheimer's dementia (AD) risk. This study aimed to evaluate the risk of AD associated with the use of sedative-hypnotics. A retrospective cohort study was conducted with randomly selected 5% samples from ≥50 years old beneficiaries of National Health Insurance Service (NHIS) of Korea from January 2002 to December 2015. The exposure to sedative-hypnotics was defined when prescribed over 30 defined daily dose (DDD) after January 2004 and it was categorized by prescribed dosage, types and half-lives of benzodiazepines. Time-dependent Cox regression model with a lag period of 5-years was used to evaluate the association between use of sedative-hypnotics and the risk of subsequent AD. Sensitivity analysis was performed for restricting sedative-hypnotics only when prescribed with insomnia. A total of 268,170 subjects were identified and subjects exposed to sedative-hypnotics showed a higher risk of AD (HR: 1.79; 95% CI: 1.72–1.86) than those who were not. There was an increased risk of AD among subjects exposed to benzodiazepines or zolpidem (HR: 1.75; 95% CI: 1.67–1.82) and antidepressants or low-dose antipsychotics (HR: 1.63; 95% CI: 1.42–1.87). The risk of AD was increased regardless of dose of sedative-hypnotics and half-life among benzodiazepines, especially in exposure to more than 360 DDD of sedative-hypnotics (HR: 1.78; 95% CI: 1.60–1.99) and the long-acting benzodiazepine (HR:1.77; 95% CI: 1.65–1.89).

Introduction

Hypnotics are widely prescribed for controlling insomnia. There has been an emerging issue about the health outcomes of hypnotics use because of the high prescription rate not only in Korea but also worldwide, especially among elderly people [1–3]. Among the issues related to

approval, the researchers can receive the data with a certain fee. The authors did not have any special access privileges to the database.

Funding: This work was supported by the research fund of Mental Health Technology Development Project (Project No. HM15C1197), (http://www.mhrnd.re.kr/). The funders had no role in study design, data collection and analysis, decision to publish, or preparation of the manuscript.

Competing interests: The authors have declared that no competing interests exist.

the risks and safety of hypnotics [4–7], dementia has been of interest because of the side effect of hypnotics having sedative potent such as benzodiazepines (BZDs) [8, 9].

Dementia is becoming an issue for public health because the number of dementia patients is growing rapidly worldwide in ageing societies [10, 11]. There were approximately 47 million dementia patients in 2016, and there are expected to be 131 million worldwide in 2050 [11]. In Korea, the prevalence of dementia over 65 years old was reported to be 9.2%, and this rate was higher than in Western and other Asian countries [12]. Furthermore, the burden of disease due to dementia was 528 disability-adjusted life years (DALYs) per 100,000 in 2008 in Korea, which is expected triple by 2050 [13, 14].

There have been observational studies reporting the association between hypnotics use and the risk of dementia [15–26]. Although some studies have reported that there is an increased risk of dementia with hypnotics use [18–26], others have reported that there is no association [15–17]. A meta-analysis conducted in 2015 reported that long-term use of BZDs increased the risk of dementia [27]. However, they commented that there might be the limitation of possible reverse causation, and a study with a longer follow-up period is needed.

Korea has the National Health Insurance Service (NHIS), which is a single medical insurance claim system. NHIS, which covers over 97% of the Korean population, collects individuals' demographic factors as well as all medical claim data such as disease diagnosis and drug prescriptions from every resident in Korea.

In this study, we hypothesized that brain hypoactivity or cognitive impairment caused by sedative effect of hypnotics could precipitate the development of dementia. However, pathophysiology of dementia differed by type and vascular or anatomical effect of sedative hypnotics on brain was less likely, so we would focus on the risk of Alzheimer's dementia. Therefore, we investigated the association between sedative-hypnotic use and the risk of Alzheimer's dementia using the NHIS database representative of the Korean population.

Methods

Data source, study population

This study was approved by the Institutional Review Board of Seoul National University Hospital (H-1508-031-694). We designed a retrospective cohort study with the NHIS data from January 2002 to December 2015, which comprised a randomly selected sample of 5% of NHIS beneficiaries over 50 years old. People who died, were lost to follow-up, diagnosed with any type of dementia, or had prescribed any sedative-hypnotics before January 2004 were excluded. The patients excluded by diagnosis of dementia were defined using the International Classification of Disease 10th Revision (ICD-10), which included Alzheimer's dementia [F00, G30], vascular dementia [F01], and other types of dementia [F02, F03, G31, G32].

Exposure definition

The sedative-hypnotics used in this study included BZDs and zolpidem. In addition, antidepressants and low-dose formulation antipsychotics as off-label prescribed hypnotic drugs in Korea were also included. These drugs have sedative potent and widely prescribed for insomnia in Korea. These study drugs are listed on S1 Appendix.

We tracked the prescription of these sedative-hypnotics from January 2004 and determined the accumulated dosage. The dosage of sedative-hypnotics was standardized by defined daily dose (DDD), which is a statistical measure of drug consumption suggested by the World Health Organization (WHO) [28]. We classified the subjects as exposed after the time when cumulative dosage of sedative-hypnotics exceeded 30 DDD, which is the assumed average maintenance dose for 30 days used for its main indication in adults. In addition, to consider

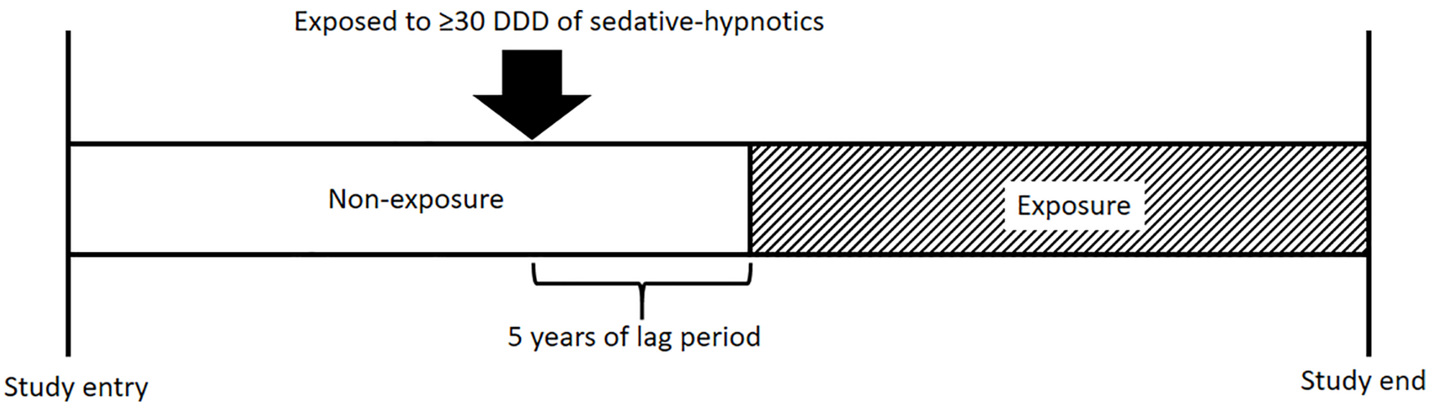

Fig 1. Description of follow-up of study population.

https://doi.org/10.1371/journal.pone.0204413.g001

the possible reverse causality of Alzheimer's dementia [29–31], a 5-year of lag period was applied after the date of exposure. Thus, as described in Fig 1, the subject was followed-up as non-exposed until 5 years after being prescribed more than 30 DDD of sedative-hypnotics and then considered as exposed thereafter.

We also classified the exposures by prescribed dosage, types, and half-lives among BZDs. We categorized the prescribed dosage of sedative-hypnotics into three groups, which were 30 to 179 DDD, 180 to 359 DDD, and over 360 DDD. In addition, we classified sedative-hypnotics into two classes based on the mechanism—gamma-aminobutyric acid A ($GABA_A$) receptor agonists (BZDs and zolpidem) and other drugs (sedative antidepressants and sedative antipsychotics). We calculated the accumulated dosage of each class of sedative-hypnotics separately and considered subjects to be exposed when each prescribed dosage exceeded 30 DDD. If subjects were exposed to both $GABA_A$ receptor agonists and other drugs, the subjects were categorized as being exposed to both classes of sedative-hypnotics. Classification by half-life was considered only among BZD drugs. We classified BZDs into short-acting BZDs (<3 hours), intermediate-acting BZDs (3–20 hours), and long-acting BZDs (>20 hours) based on their half-lives. If a patient was exposed more than two BZDs with different half-lives, he or she was considered to be exposed to longer-acting BZDs. When analysing by BZDs' half-life, patients who were exposed to zolpidem, antidepressants, or low-dose antipsychotics were treated as censored after the date of exposure.

Study endpoint

The study population was followed up until the diagnosis of Alzheimer's dementia, loss of follow-up, or 31 December 2015. The diagnosis of Alzheimer's dementia was defined as the first date of claim data with diagnostic code of F00 or G30 after January 2004.

Covariates

Known risk factors for Alzheimer's dementia such as diabetes, hypertension, hyperlipidaemia, and cerebrovascular disease were considered to be potential confounders [10, 32–34]. Furthermore, sedative-hypnotics are commonly prescribed for treatment of insomnia as well as psychiatric disorders such as anxiety disorders, depression, and psychotic disorders [35]. In addition, these psychiatric disorders are known for risk factors and prodromal symptoms of Alzheimer's dementia [29]. We extracted these potential confounders using ICD-10. Diabetes mellitus [E10-E14], hypertension [I10], hyperlipidaemia [E78], and cerebrovascular disease

(CVD) [I60-I69] were included if the patient had at least two diagnoses on claim per year from January 2002 to the endpoint. Anxiety disorders [F4x], depression [F32, F33], insomnia [F510, G470], and psychotic disorders [F2x] were included if he or she had at least two diagnoses on claim per year from January 2004 to the endpoint, because these diseases could be related to sedative-hypnotics use. In NHIS, the insurance premium is determined by the income level of individuals [36]. Therefore, we also extracted insurance premium information of the first year of the study period and classified it into three groups based on their levels to reflect socioeconomic status.

Statistical analysis

We compared the characteristics between the ever-exposed, who were prescribed over 30 DDD of sedative-hypnotics during the study period, and the non-exposed, who were not; we used the Chi-square test for categorical values and the t-test for continuous values to compare both groups. We set an exposure status as time-varying covariate, so that the variable might be changed by their usage of sedative-hypnotics during the study period.

We used a time-dependent Cox regression model and counting process model to consider time-dependent bias and to compute hazard ratio (HR) and 95% confidence interval (CI) [37–39]. Because Alzheimer's dementia is age dependent disease, age was chosen as the time scale in the time-dependent Cox regression model [40]. We presented two adjusted models: the first model was adjusted for sex, diabetes mellitus, hypertension, hyperlipidaemia, CVD, and medical insurance premium, and the second model was additionally adjusted for anxiety disorders, depression, insomnia, and psychotic disorders. All statistical analyses were performed with SAS 9.4 (SAS Institute Inc., Cary, NC).

Sensitivity analysis

We restricted the sedative-hypnotics only when prescribed with the diagnostic code of insomnia [F510, G470]. We also included the exposure to sedative-hypnotics without the diagnostic code of insomnia into the model as a separate category. To compare the risk of Alzheimer's dementia between never users who prescribed none of any sedative-hypnotics and users who prescribed at least one sedative-hypnotics, we categorized the prescribed dosage of sedative-hypnotics into four groups, which were none, 1 to 29 DDD, 30 to 179 DDD, 180 to 359 DDD, and over 360 DDD.

Insomnia could be a presenile appearance of Alzheimer's dementia. Thus, we additionally analyzed the association between sedative-hypnotics use and Alzheimer's dementia among subjects who had insomnia.

In these sensitivity analysis, insomnia was not adjusted in the Cox model.

Results

We identified 486,622 subjects, who were randomly selected 5% of a sample of Korean NHIS beneficiaries aged 50 years and over, between January 2002 and December 2015. Of these, subjects who died or lost follow-up, had diagnosed with any type of dementia, and had used of any sedative-hypnotics between January 2002 and December 2003 were excluded. In addition, subjects who had invalid data were excluded. Finally, a total of 268,170 subjects remained for the analyses. Of these, the mean follow-up time was 10.97 years, and 27,925 patients developed Alzheimer's dementia during the study period (Fig 2).

Characteristics of the study population between non-exposed and ever-exposed to sedative-hypnotics are shown in Table 1. The ever-exposed to sedative-hypnotics group was more likely to be female and have comorbidities such as diabetes mellitus, hypertension, hyperlipidaemia,

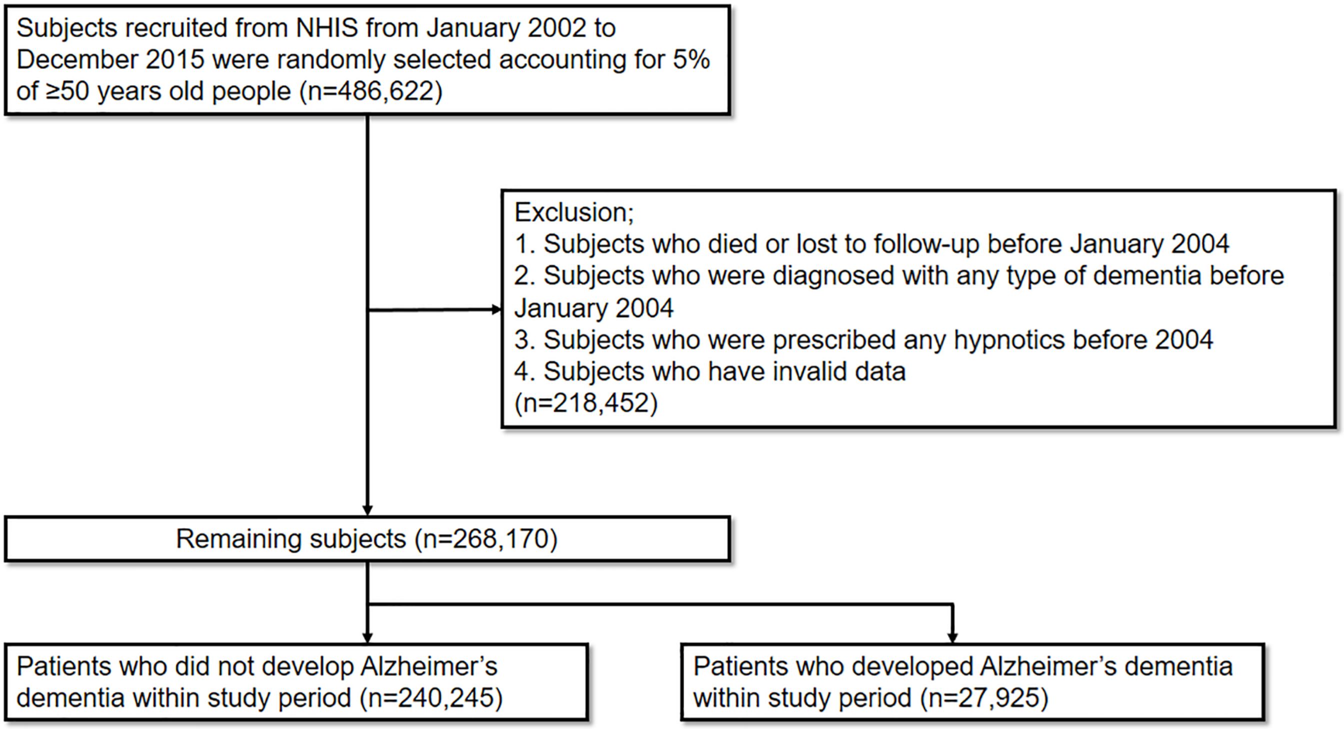

Fig 2. Selection of subjects included in the study.

https://doi.org/10.1371/journal.pone.0204413.g002

Table 1. Characteristics of study population between sedative-hypnotics exposure and non-exposure.

	Total N = 268,170	Non-exposed N = 233,581	Ever-exposed N = 34,589	p-value*
**Age† (mean (SD))**	61.12 (9.12)	61.15 (9.32)	60.93 (7.64)	<0.01
**Sex**				
**Male**	146,663 (54.69)	131,515 (56.30)	15,148 (43.79)	<0.01
**Female**	121,507 (45.31)	102,066 (43.70)	19,441 (56.21)	
**Insurance premium**				
**Low**	96,159 (35.86)	81,661 (34.96)	14,498 (41.92)	<0.01
**Middle**	87,446 (32.61)	77,229 (33.06)	10,217 (29.54)	
**High**	84,565 (31.53)	74,691 (31.98)	9,874 (28.55)	
**Comorbidities**				
**Diabetes mellitus**	52,715 (19.66)	43,503 (18.62)	9,212 (26.63)	<0.01
**Hypertension**	122,410 (45.65)	101,024 (43.25)	21,386 (61.83)	<0.01
**Hyperlipidemia**	56,049 (20.90)	43,812 (18.76)	12,237 (35.38)	<0.01
**Cerebrovascular disease**	22,352 (8.34)	17,414 (7.46)	4,938 (14.28)	<0.01
**Insomnia**	7,862 (2.93)	3,942 (1.69)	3,920 (11.33)	<0.01
**Anxiety disorders**	16,988 (6.33)	8,151 (3.49)	8,837 (25.55)	<0.01
**Depression**	7,784 (2.90)	4,301 (1.84)	3,483 (10.07)	<0.01
**Psychotic disorders**	1,647 (0.61)	1,413 (0.60)	234 (0.68)	0.11

*p-value was calculated by Chi-square and t-tests comparing hypnotics exposure and non-exposure

†Age at study entry

https://doi.org/10.1371/journal.pone.0204413.t001

Table 2. Hazard ratios of use of sedative-hypnotics on Alzheimer's dementia by time-dependent Cox regression model classified by dose, types and half-lives among benzodiazepines.

	Person-years	Number of events	Crude HR	95% CI	Adjusted HR*	95% CI	Adjusted HR†	95% CI
<30 DDD of any sedative-hypnotics	2,839,761	24,590	1.00	Reference	1.00	Reference	1.00	Reference
≥30 DDD of any sedative-hypnotics	102,327	3,335	1.92	(1.84–1.99)	1.89	(1.82–1.96)	1.79	(1.72–1.86)
<30 DDD of any sedative-hypnotics	2,839,761	24,590	1.00	Reference	1.00	Reference	1.00	Reference
30–179 DDD of any sedative-hypnotics	80,439	2,371	1.69	(1.61–1.77)	1.69	(1.61–1.77)	1.64	(1.56–1.72)
180–359 DDD of any sedative-hypnotics	12,575	526	1.78	(1.60–1.97)	1.71	(1.54–1.90)	1.60	(1.44–1.78)
≥360 DDD of any sedative-hypnotics	9,312	438	2.15	(1.93–2.39)	2.02	(1.82–2.25)	1.78	(1.60–1.99)
<30 DDD of GABAA‡ and other drugs§	2,842,491	24,668	1.00	Reference	1.00	Reference	1.00	Reference
≥30 DDD of GABAA	85,611	2,727	1.83	(1.75–1.91)	1.82	(1.74–1.89)	1.75	(1.67–1.82)
≥30 DDD of other drugs	8,116	242	1.83	(1.60–2.10)	1.74	(1.52–2.00)	1.63	(1.42–1.87)
≥30 DDD of GABAA and ≥30 DDD of other drugs	5,870	288	2.61	(2.29–2.97)	2.45	(2.15–2.78)	2.02	(1.77–2.30)
<30 DDD of benzodiazepines	2,852,245	24,935	1.00	Reference	1.00	Reference	1.00	Reference
≥30 DDD of short-acting benzodiazepines	11,608	314	1.53	(1.36–1.73)	1.56	(1.38–1.76)	1.53	(1.35–1.73)
≥30 DDD of intermediate-acting benzodiazepines	23,211	716	1.74	(1.60–1.89)	1.71	(1.57–1.86)	1.62	(1.49–1.76)
≥30 DDD of long-acting benzodiazepines	30,293	1,035	1.85	(1.73–1.98)	1.82	(1.70–1.95)	1.77	(1.65–1.89)

Defined daily dose; DDD

*adjusted for sex, diabetes mellitus, hypertension, hyperlipidaemia, cerebrovascular disease, insurance premium

†adjusted for sex, diabetes mellitus, hypertension, hyperlipidaemia, cerebrovascular disease, insurance premium, anxiety, insomnia, depression, psychotic disorder

‡benzodiazepines or zolpidem

§antidepressants or low-dose antipsychotics

https://doi.org/10.1371/journal.pone.0204413.t002

CVD, insomnia, anxiety disorders, and depression. The ever-exposed group was more likely to have lower income level than the non-exposed. The hazard ratios of these covariates were presented on S1 Table.

Table 2 shows the association between sedative-hypnotics use and the risk of Alzheimer's dementia. The risk of Alzheimer's dementia was significantly higher in for those who were prescribed over 30 DDD of any sedative-hypnotic (HR: 1.79, 95% CI: 1.72–1.86) than those who were not exposed, after adjustment. Subjects exposed to over 360 DDD showed the greatest elevated risk of Alzheimer's dementia (HR: 1.78, 95% CI: 1.60–1.99), while the hazard of subjects with 30 to 179 DDD exposure (HR: 1.64, 95% CI: 1.56–1.72) and 180 to 359 DDD exposure (HR: 1.60, 95% CI: 1.44–1.78) also increased, although they did not show a typical dose-response relationship. When considering the type of drug, we found that $GABA_A$ receptor agonist exposure was related to a higher risk of Alzheimer's dementia (HR: 1.75, 95% CI: 1.67–1.82) than exposure to other types including antidepressants or antipsychotics (HR:1.63, 95% CI: 1.42–1.87), but exposure to both classes showed the highest risk (HR: 2.02, 95% CI: 1.77–2.30) compared to non-exposure. When classifying by BZD half-life, we found that BZD exposure was significantly associated with Alzheimer's dementia regardless of half-life. The HR of

Table 3. Hazard ratios of use of sedative-hypnotics prescribed in insomnia patients on Alzheimer's dementia by time-dependent Cox regression model classified by dose, types and half-lives among benzodiazepines.

	Person-years	Number of events	Crude HR	95% CI	Adjusted HR*	95% CI	Adjusted HR†	95% CI
<30 DDD* of any sedative-hypnotics	2,839,761	24,590	1.00	Reference	1.00	Reference	1.00	Reference
≥30 DDD of any sedative-hypnotics w/o insomnia	88,313	2,732	1.83	(1.75–1.91)	1.81	(1.73–1.89)	1.73	(1.66–1.81)
≥30 DDD of any sedative-hypnotics w/ insomnia	14,013	603	2.16	(1.97–2.36)	2.12	(1.94–2.32)	1.94	(1.77–2.14)
<30 DDD of any sedative-hypnotics	2,839,761	24,590	1.00	Reference	1.00	Reference	1.00	Reference
≥30 DDD of any sedative-hypnotics w/o insomnia	88,313	2,732	1.83	(1.75–1.91)	1.81	(1.73–1.89)	1.73	(1.66–1.81)
30–179 DDD of any sedative-hypnotics w/ insomnia	10,802	447	2.00	(1.80–2.23)	1.98	(1.78–2.21)	1.84	(1.65–2.05)
180–359 DDD of any sedative-hypnotics w/ insomnia	1,818	73	1.43	(1.08–1.91)	1.39	(1.05–1.86)	1.29	(0.97–1.73)
≥360 DDD of any sedative-hypnotics w/ insomnia	1,393	83	2.55	(1.99–3.28)	2.43	(1.89–3.12)	2.10	(1.63–2.72)
<30 DDD of GABAA‡ or other drugs§	2,839,761	24,590	1.00	Reference	1.00	Reference	1.00	Reference
≥30 DDD of any sedative-hypnotics w/o insomnia	88,545	2,740	1.83	(1.76–1.91)	1.81	(1.73–1.89)	1.73	(1.66–1.81)
≥30 DDD of GABAA w/ insomnia	13,025	561	2.13	(1.94–2.34)	2.11	(1.92–2.31)	1.95	(1.77–2.15)
≥30 DDD of other drugs w/ insomnia	126	4	1.86	(0.60–5.78)	1.63	(0.53–5.05)	1.42	(0.46–4.40)
≥30 DDD of GABAA and ≥30 DDD of other drugs w/ insomnia	630	30	2.27	(1.49–3.44)	2.07	(1.37–3.15)	1.60	(1.05–2.43)
<30 DDD of benzodiazepines	2,839,761	24,590	1.00	Reference	1.00	Reference	1.00	Reference
≥30 DDD of any benzodiazepines w/o insomnia	73,157	2,197	1.75	(1.67–1.84)	1.74	(1.66–1.83)	1.69	(1.61–1.77)
≥30 DDD of short-acting benzodiazepines	2,221	106	2.04	(1.64–2.55)	2.05	(1.64–2.56)	2.00	(1.60–2.50)
≥30 DDD of intermediate-acting benzodiazepines	1,424	70	2.25	(1.72–2.94)	2.18	(1.67–2.86)	2.01	(1.53–2.64)
≥30 DDD of long-acting benzodiazepines	794	37	2.06	(1.43–2.97)	1.98	(1.37–2.84)	1.82	(1.26–2.62)

Defined daily dose; DDD

*adjusted for sex, diabetes mellitus, hypertension, hyperlipidaemia, cerebrovascular disease, insurance premium

†adjusted for sex, diabetes mellitus, hypertension, hyperlipidaemia, cerebrovascular disease, insurance premium, anxiety, depression, psychotic disorder

‡benzodiazepines or zolpidem

§antidepressants or low-dose antipsychotics

https://doi.org/10.1371/journal.pone.0204413.t003

short-acting BZDs was 1.53 (95% CI: 1.35–1.73), intermediate-acting BZDs was 1.62 (95% CI: 1.49–1.76), and long-acting BZDs was 1.77 (95% CI: 1.65–1.89), after adjustment.

In sensitivity analysis (Table 3), the HRs showed similar trends as the main analysis. The risk of Alzheimer's dementia was significantly higher regardless of the classification of sedative-hypnotics. Patients who were exposed to sedative-hypnotics without the diagnostic code of insomnia showed a significantly higher risk of Alzheimer's dementia (HR: 1.73, 95% CI: 1.66–1.81), and the risk was also high for exposure with insomnia (HR: 1.94, 95% CI: 1.77–2.14). The risk of Alzheimer' dementia was significantly elevated in $GABA_A$ receptor agonist exposure (HR: 1.95, 95% CI: 1.77–2.15) and exposure to both classes (HR: 1.60, 95% CI: 1.05–2.43), but not in other classes of exposure (HR: 1.42, 95% CI: 0.46–4.40). Subjects who were

exposed to sedative-hypnotics with diagnostic code of insomnia for over 360 DDD had the highest risk of Alzheimer's dementia (HR: 2.10, 95% CI: 1.63–2.72), which was similar to the main analysis. Among BZD exposures, intermediate-acting BZDs showed the highest risk of Alzheimer's dementia (HR: 2.01, 95% CI: 1.53–2.64).

Among subjects who had insomnia diagnosis (n = 7,862), the association between use of sedative-hypnotics and Alzheimer's dementia was similar to that of the main analysis (S2 Table). Exposure to sedative-hypnotics showed increased risk of Alzheimer's dementia (HR: 2.31, 95% CI: 2.06–2.59), especially subjects exposed to over 360 DDD of sedative-hypnotics exposure (HR: 2.50, 95% CI: 2.07–3.02). The risk of Alzheimer's dementia was also elevated in subjects who prescribed at least one tablet of sedative-hypnotics (S3 Table). Hazard ratios of subjects who exposed 1 to 29 DDD, 30 to 179 DDD, 180 to 359 DDD and over 360 DDD were 1.54 (95% CI: 1.50–1.58), 1.92 (95% CI: 1.83–2.01), 1.89 (95% CI: 1.71–2.10), and 2.12 (95% CI: 1.89–2.36) respectively compared to never users.

Discussion

In our retrospective cohort study from the National Health Insurance Database of Korea with a mean follow-up period of 10.97 years, the risk of Alzheimer's dementia was significantly associated with sedative-hypnotics use regardless of prescribed dosage, drug type, or half-life of benzodiazepine.

We excluded subjects who were prescribed any sedative-hypnotics before 2004, which led to approximately 55% of the total subjects remaining. This may also provide evidence that the prevalence of prescription of sedative-hypnotics was high at approximately 45% for the Korean population over 50 years old who were prescribed at least one sedative-hypnotic.

In previous studies, the association between benzodiazepines or Z-drugs ($GABA_A$ receptor agonists) and the risk of dementia has been reported, and our findings support these results [18–27]. A meta-analysis including six studies reported that benzodiazepine users showed increased risk of dementia compared to never-users (RR: 1.49, 95% CI: 1.30–1.72) [27]. Although our study did not show typical dose-response relationships after adjusting psychiatric disorders, the risk was elevated dose-dependently in the other model adjusted for other comorbities and it was markedly high in high dosage group who were exposed to over 360 DDD of sedative-hypnotics in all model. This aspect was also shown in other previous studies [20, 22, 24, 25]. There have been few studies about the association between the risk of Alzheimer's dementia and antidepressant or antipsychotics. A meta-analysis on the association between antidepressant and Alzheimer's disease was conducted in 2017 and included 5 studies. It reported that use of antidepressants was associated with increased risk of Alzheimer's disease (OR: 2.17, 95% CI: 1.41–3.33) [41], which is consistent with the current results. On the matter of the half-life of BZDs, the risk of Alzheimer's dementia was elevated regardless of half-life and it was higher in the exposed to longer-acting BZDs. These results were consistent with other previous studies which had done the analysis classified by half-life [24, 25].

We also conducted the sensitivity analyses with subjects with diagnostic code of insomnia to rule out the possibility that the observed association was due to insomnia as an early manifestation of Alzheimer's dementia. The strengths of risk were somewhat higher than in the main analysis and the results showed similar trends to the main analysis except for antidepressants or antipsychotics. A study using Taiwan's National Health Insurance Database restricted analysis to subjects with insomnia [25], which was similar design with our sensitivity analysis. In their results, the risk of dementia was elevated with more than 30 DDD of hypnotics exposure (HR: 2.34, 95% CI: 1.92–2.85), while our corresponding results showed HR of 2.22 (95% CI: 1.97–2.51).

The mechanism of how sedative-hypnotics could increase the risk of Alzheimer's dementia is still unclear. Pariente [42] hypothesized that as benzodiazepines lower brain activity, the patients' capacity to neurally compensate and have a cognitive reserve could be limited, thus precipitating dementia. Otherwise, patients with lesions of dementia without any symptoms have increased risk of becoming symptomatic with benzodiazepines because astrocytes in amyloid plaques have GABA-secreting activity. Another possible explanation is reverse causality and that insomnia could be a prodromal symptom of Alzheimer's disease [29, 30]. However, in our study, we set a 5-year lag period between sedative-hypnotic exposure and diagnosis of the disease to rule out reverse causality.

In our study, we had several strengths compared to previous studies. First, our study population comprised 268,170 subjects, which was a large enough sample to secure statistical power. Second, we used nationally representative data so that it could represent the general population of people over 50 years old in Korea. In addition, the development of Alzheimer's dementia takes a long time [31], and our study had a maximum of 12 years and an average of 10.97 years of follow-up period, which is sufficient time to develop Alzheimer's dementia after exposure to sedative-hypnotics. Third, the other previous studies only targeted hypnotics specific to benzodiazepine or Z-drugs that target GABA receptors. Our study covers $GABA_A$ receptor agonists as well as antidepressants and low-dose antipsychotics, and we calculated the risk of each drug exposure separately. Furthermore, we categorized sedative-hypnotics in this way so that it is possible to compare the risks of Alzheimer's dementia by each drug. To our knowledge, this spectrum of sedative-hypnotics has not yet been studied. Lastly, time-dependent modelling used in this analysis could statistically control for immortal time bias and length bias.

However, there were also limitations in our study. Although we set a 5-year lag period after the time when exposed over 30 DDD of sedative-hypnotics, the possibility of reverse causality could not be totally excluded. Because the prodromal symptoms of dementia can occur more than 10 years before symptomatic dementia, studies with longer lag periods than ours are needed [29, 31]. Other limitations arose from the characteristics of insurance claim data. The NHIS database we used did not include lifestyle factors such as smoking and alcohol consumption, so these possible confounding factors could not be considered in our study. In addition, we did not have the results of diagnostic evaluations on Alzheimer's dementia such as mental status examination. Also, defining Alzheimer's dementia with a single diagnosis code could overestimate the prevalence. Therefore, the validity of this study's definition of Alzheimer's dementia might be challenged. However, the potential misclassification of Alzheimer's dementia, unlikely differed by prescription of sedative-hypnotics, could make the results move toward null. Also we found that the incidence rate of Alzheimer's dementia in our study was 8.74 per 1,000 person-years, which was similar to a previous study conducted in Korea (overall incidence: 7.1 per 1,000 person-years, 95% CI: 5.2–9.7) [43]. Another limitation was that prescription of drugs was different from administration of drugs this may have overestimated the exposure to sedative-hypnotics. This was an inevitable limitation when using claim data without the information about actual drug administration. Lastly, each patient's indication for sedative-hypnotics was uncertain. For example, if patients were prescribed benzodiazepines with diagnostic codes of both insomnia and anxiety disorders, the reason that patients were prescribed benzodiazepines could not be determined. For this reason, we did the sensitivity analysis with exposure limited to insomnia, but such a limitation could not be totally excluded.

Conclusion

The risk of Alzheimer's dementia was elevated when exposed to sedative-hypnotics regardless of drug types in Korean patients 50 years old or older; therefore, sedative-hypnotics need to be prescribed with caution. However, other confounding factors such as smoking status and alcohol consumption could not be controlled in our study. A more precise result could be obtained when controlling for all possible confounding factors, indicating that a prospective cohort study that can control for all possible factors needs to be conducted.

Supporting information

S1 Appendix. The list of study drugs in this study.
(DOCX)

S1 Table. The hazard ratios of all covariates on the risk of Alzheimer's dementia.
(DOCX)

S2 Table. Hazard ratios of use of sedative-hypnotics on Alzheimer's dementia among subjects who had insomnia.
(DOCX)

S3 Table. The hazard ratios of use of sedative-hypnotics on Alzheimer's dementia based on dosage.
(DOCX)

Acknowledgments

This work was supported by research fund of Mental Health Technology Development Project (Project No. HM15C1197). The funding sources had no involvement in the study, data analysis, and interpretation.

Author Contributions

Conceptualization: Joonki Lee, Sun Jae Jung, Jae-won Choi, Aesun Shin, Yu Jin Lee.

Data curation: Joonki Lee, Sun Jae Jung.

Formal analysis: Joonki Lee.

Funding acquisition: Yu Jin Lee.

Investigation: Joonki Lee, Sun Jae Jung, Jae-won Choi, Aesun Shin.

Methodology: Joonki Lee, Sun Jae Jung, Aesun Shin, Yu Jin Lee.

Supervision: Aesun Shin, Yu Jin Lee.

Writing – original draft: Joonki Lee.

Writing – review & editing: Joonki Lee, Aesun Shin, Yu Jin Lee.

References

1. Oh S-H, Oh KS, Lee K-U, Woo J-M, Lee B-C, Hwang JS, et al. In-depth investigation for prescribing trends of benzodiazepines in South Korea. Int J Clin Pharmacol Ther. 2014; 52(6):460–70. Epub 2014/05/03. https://doi.org/10.5414/CP202008 PMID: 24786014.
2. Chung S, Park B, Yi K, Lee J. Pattern of Hypnotic Drug Prescription in South Korea: Health Insurance Review and Assessment Service-National Patients Sample. Sleep Medicine Research. 2013; 4(2):51–5. doi: s. PubMed PMID: SC000006407.

3. Kaufmann CN, Spira AP, Alexander GC, Rutkow L, Mojtabai R. Trends in prescribing of sedative-hypnotic medications in the USA: 1993–2010. Pharmacoepidemiology and Drug Safety. 2016; 25(6):637–45. Epub 2015/12/30. https://doi.org/10.1002/pds.3951 PMID: 26711081; PubMed Central PMCID: PMCPMC4889508.
4. Kripke DF. Chronic hypnotic use: deadly risks, doubtful benefit. REVIEW ARTICLE. Sleep medicine reviews. 2000; 4(1):5–20. Epub 2003/01/18. https://doi.org/10.1053/smrv.1999.0076 PMID: 12531158.
5. Buscemi N, Vandermeer B, Friesen C, Bialy L, Tubman M, Ospina M, et al. The Efficacy and Safety of Drug Treatments for Chronic Insomnia in Adults: A Meta-analysis of RCTs. Journal of General Internal Medicine. 2007; 22(9):1335–50. Epub 2007/07/11. https://doi.org/10.1007/s11606-007-0251-z PubMed PMID: PMC2219774; PubMed Central PMCID: PMCPMC2219774. PMID: 17619935
6. Yang KI, Hwangbo Y. The Efficacy and Safety of Long-Term Use of Hypnotics. J Korean Sleep Res Soc. 2010; 7:25–31.
7. Kripke DF. Risks of chronic hypnotic use. Sleep and sleep disorders. 2006:141–5.
8. Vermeeren A, Coenen AML. Chapter 5—Effects of the use of hypnotics on cognition. In: Van Dongen HPA, Kerkhof GA, editors. Progress in Brain Research. 190: Elsevier; 2011. p. 89–103.
9. Buffett-Jerrott SE, Stewart SH. Cognitive and Sedative Effects of Benzodiazepine Use. Current Pharmaceutical Design. 2002; 8(1):45–58. http://dx.doi.org/10.2174/1381612023396654. PMID: 11812249
10. Sosa-Ortiz AL, Acosta-Castillo I, Prince MJ. Epidemiology of Dementias and Alzheimer's Disease. Archives of Medical Research. 2012; 43(8):600–8. Epub 2012/11/20. https://doi.org/10.1016/j.arcmed.2012.11.003 PMID: 23159715.
11. Prince M, Comas-Herrera A, Knapp M, Guerchet M, Karagiannidou M. World Alzheimer report 2016: improving healthcare for people living with dementia: coverage, quality and costs now and in the future. 2016.
12. Kim YJ, Han JW, So YS, Seo JY, Kim KY, Kim KW. Prevalence and Trends of Dementia in Korea: A Systematic Review and Meta-Analysis. J Korean Med Sci. 2014; 29(7):903–12. Epub 2014/07/22. https://doi.org/10.3346/jkms.2014.29.7.903 PMID: 25045221; PubMed Central PMCID: PMCPMC4101777.
13. Metrics IfH, Evaluation. GBD compare. IHME, University of Washington Seattle^ eWA WA; 2015.
14. Park J-H, Eum J-H, Bold B, Cheong H-K. Burden of disease due to dementia in the elderly population of Korea: present and future. BMC Public Health. 2013; 13(1):293. Epub 2013/04/05. https://doi.org/10.1186/1471-2458-13-293 PMID: 23552095; PubMed Central PMCID: PMCPMC3626581.
15. Imfeld P, Bodmer M, Jick SS, Meier CR. Benzodiazepine Use and Risk of Developing Alzheimer's Disease or Vascular Dementia: A Case–Control Analysis. Drug Safety. 2015; 38(10):909–19. Epub 2015/07/01. https://doi.org/10.1007/s40264-015-0319-3 PMID: 26123874.
16. Gray SL, Dublin S, Yu O, Walker R, Anderson M, Hubbard RA, et al. Benzodiazepine use and risk of incident dementia or cognitive decline: prospective population based study. BMJ. 2016; 352:i90. Epub 2016/02/04. https://doi.org/10.1136/bmj.i90 PMID: 26837813; PubMed Central PMCID: PMCPMC4737849.
17. Fastbom J, Forsell Y, Winblad B. Benzodiazepines May Have Protective Effects Against Alzheimer Disease. Alz Dis Assoc Dis. 1998; 12(1):14–7. https://doi.org/10.1097/00002093-199803000-00002 PubMed PMID: 00002093-199803000-00002.
18. Lagnaoui R, Bégaud B, Moore N, Chaslerie A, Fourrier A, Letenneur L, et al. Benzodiazepine use and risk of dementia: a nested case–control study. J Clin Epidemiol. 2002; 55(3):314–8. PMID: 11864804
19. Gallacher J, Elwood P, Pickering J, Bayer A, Fish M, Ben-Shlomo Y. Benzodiazepine use and risk of dementia: evidence from the Caerphilly Prospective Study (CaPS). J Epidemiol Community Health. 2012; 66(10):869–73. Epub 2011/10/29. https://doi.org/10.1136/jech-2011-200314 PMID: 22034632.
20. Wu CS, Wang SC, Chang IS, Lin KM. The association between dementia and long-term use of benzodiazepine in the elderly: nested case-control study using claims data. The American journal of geriatric psychiatry: official journal of the American Association for Geriatric Psychiatry. 2009; 17(7):614–20. Epub 2009/06/24. https://doi.org/10.1097/JGP.0b013e3181a65210 PMID: 19546656.
21. de Gage SB, Bégaud B, Bazin F, Verdoux H, Dartigues J-F, Pérès K, et al. Benzodiazepine use and risk of dementia: prospective population based study. Bmj. 2012; 345:e6231. https://doi.org/10.1136/bmj.e6231 PMID: 23045258
22. Billioti de Gage S, Moride Y, Ducruet T, Kurth T, Verdoux H, Tournier M, et al. Benzodiazepine use and risk of Alzheimer's disease: case-control study. BMJ: British Medical Journal. 2014; 349:g5205. Epub 2014/09/12. https://doi.org/10.1136/bmj.g5205 PMID: 25208536; PubMed Central PMCID: PMCPMC4159609.

23. Wu C-S, Ting T-T, Wang S-C, Chang I-S, Lin K-M. Effect of benzodiazepine discontinuation on dementia risk. The American Journal of Geriatric Psychiatry. 2011; 19(2):151–9. Epub 2010/09/03. https://doi.org/10.1097/JGP.0b013e3181e049ca PMID: 20808131.

24. Gomm W, von Holt K, Thomé F, Broich K, Maier W, Weckbecker K, et al. Regular Benzodiazepine and Z-Substance Use and Risk of Dementia: An Analysis of German Claims Data. Journal of Alzheimer's Disease. 2016; 54(2):801–8. Epub 2016/08/29. https://doi.org/10.3233/JAD-151006 PubMed PMID: WOS:000384087200033. PMID: 27567804

25. Chen P-L, Lee W-J, Sun W-Z, Oyang Y-J, Fuh J-L. Risk of Dementia in Patients with Insomnia and Long-term Use of Hypnotics: A Population-based Retrospective Cohort Study. PloS one. 2012; 7(11): e49113. Epub 2012/11/13. https://doi.org/10.1371/journal.pone.0049113 PubMed PMID: PMC3492301; PubMed Central PMCID: PMCPMC3492301. PMID: 23145088

26. Shash D, Kurth T, Bertrand M, Dufouil C, Barberger-Gateau P, Berr C, et al. Benzodiazepine, psychotropic medication, and dementia: A population-based cohort study. Alzheimers Dement. 2016; 12 (5):604–13. Epub 2015/11/26. https://doi.org/10.1016/j.jalz.2015.10.006 PMID: 26602630.

27. Zhong G, Wang Y, Zhang Y, Zhao Y. Association between Benzodiazepine Use and Dementia: A Meta-Analysis. PloS one. 2015; 10(5):e0127836. Epub 2015/05/29. https://doi.org/10.1371/journal.pone.0127836 PMID: 26016483; PubMed Central PMCID: PMCPMC4446315.

28. Methodology WCCfDS. Guidelines for ATC classification and DDD assignment 2013. WHO Collaborating Centre for Drug Statistics Methodology Oslo; 2012.

29. Amieva H, Le Goff M, Millet X, Orgogozo JM, Peres K, Barberger-Gateau P, et al. Prodromal Alzheimer's disease: successive emergence of the clinical symptoms. Ann Neurol. 2008; 64(5):492–8. Epub 2008/12/11. https://doi.org/10.1002/ana.21509 PMID: 19067364.

30. Barbui C, Gastaldon C, Cipriani A. Benzodiazepines and risk of dementia: true association or reverse causation? Epidemiology and psychiatric sciences. 2013; 22(04):307–8. Epub 2013/07/05. https://doi.org/10.1017/S2045796013000358 PMID: 23823009.

31. Bateman RJ, Xiong C, Benzinger TL, Fagan AM, Goate A, Fox NC, et al. Clinical and biomarker changes in dominantly inherited Alzheimer's disease. New England Journal of Medicine. 2012; 367 (9):795–804. Epub 2012/07/13. https://doi.org/10.1056/NEJMoa1202753 PMID: 22784036; PubMed Central PMCID: PMCPMC3474597.

32. Kalaria RN, Maestre GE, Arizaga R, Friedland RP, Galasko D, Hall K, et al. Alzheimer's disease and vascular dementia in developing countries: prevalence, management, and risk factors. The Lancet Neurology. 2008; 7(9):812–26. Epub 2008/08/01. https://doi.org/10.1016/S1474-4422(08)70169-8 PMID: 18667359; PubMed Central PMCID: PMCPMC2860610.

33. Luchsinger JA, Mayeux R. Cardiovascular risk factors and Alzheimer's disease. Current Atherosclerosis Reports. 2004; 6(4):261–6. https://doi.org/10.1007/s11883-004-0056-z PMID: 15191699

34. Profenno LA, Porsteinsson AP, Faraone SV. Meta-Analysis of Alzheimer's Disease Risk with Obesity, Diabetes, and Related Disorders. Biological Psychiatry. 2010; 67(6):505–12. Epub 2009/04/11. https://doi.org/10.1016/j.biopsych.2009.02.013 PMID: 19358976.

35. Fourrier A, Letenneur L, Dartigues J, Moore N, Bégaud B. Benzodiazepine use in an elderly community-dwelling population. Eur J Clin Pharmacol. 2001; 57(5):419–25. https://doi.org/10.1007/s002280100326 PubMed PMID: WOS:000171266700009. PMID: 11599660

36. Lee J, Lee JS, Park S-H, Shin SA, Kim K. Cohort Profile: The National Health Insurance Service–National Sample Cohort (NHIS-NSC), South Korea. International Journal of Epidemiology. 2017; 46(2): e15-e. https://doi.org/10.1093/ije/dyv319 PMID: 26822938

37. Andersen PK, Gill RD. Cox's Regression Model for Counting Processes: A Large Sample Study. The Annals of Statistics. 1982; 10(4):1100–20.

38. Suissa S. Immortal time bias in observational studies of drug effects. Pharmacoepidemiology and Drug Safety. 2007; 16(3):241–9. Epub 2007/01/26. https://doi.org/10.1002/pds.1357 PMID: 17252614.

39. Wolkewitz M, Allignol A, Harbarth S, de Angelis G, Schumacher M, Beyersmann J. Time-dependent study entries and exposures in cohort studies can easily be sources of different and avoidable types of bias. J Clin Epidemiol. 2012; 65(11):1171–80. https://doi.org/10.1016/j.jclinepi.2012.04.008 PubMed PMID: WOS:000309571500007. PMID: 23017635

40. Thiébaut ACM, Bénichou J. Choice of time-scale in Cox's model analysis of epidemiologic cohort data: a simulation study. Statistics in Medicine. 2004; 23(24):3803–20. Epub 2004/12/08. https://doi.org/10.1002/sim.2098 PMID: 15580597.

41. Moraros J, Nwankwo C, Patten SB, Mousseau DD. The association of antidepressant drug usage with cognitive impairment or dementia, including Alzheimer disease: A systematic review and meta-analysis. Depression and Anxiety. 2017; 34(3):217–26. Epub 2016/12/29. https://doi.org/10.1002/da.22584 PubMed PMID: PMC5347943; PubMed Central PMCID: PMCPMC5347943. PMID: 28029715

42. Pariente A, de Gage SB, Moore N, Bégaud B. The Benzodiazepine–Dementia Disorders Link: Current State of Knowledge. CNS Drugs. 2016; 30(1):1–7. Epub 2015/12/31. https://doi.org/10.1007/s40263-015-0305-4 PMID: 26715389.

43. Bae JB, Kim YJ, Han JW, Kim TH, Park JH, Lee SB, et al. Incidence of and Risk Factors for Alzheimer's Disease and Mild Cognitive Impairment in Korean Elderly. Dementia and Geriatric Cognitive Disorders. 2015; 39(1–2):105–15. Epub 2014/11/18. https://doi.org/10.1159/000366555 PMID: 25401488.
